# Nanopore sequencing improves the draft genome of the human pathogenic amoeba *Naegleria fowleri*

**DOI:** 10.1038/s41598-019-52572-0

**Published:** 2019-11-05

**Authors:** Nicole Liechti, Nadia Schürch, Rémy Bruggmann, Matthias Wittwer

**Affiliations:** 1 Interfaculty Bioinformatics Unit and Swiss Institute of Bioinformatics, University of Bern, Bern, Switzerland; 2 Spiez Laboratory, Federal Office for Civil Protection, Austrasse, Spiez Switzerland; 3 Graduate School for Cellular and Biomedical Sciences, University of Bern, Bern, Switzerland

**Keywords:** Next-generation sequencing, Parasite genomics

## Abstract

*Naegleria fowleri* is an environmental protist found in soil and warm freshwater sources worldwide and is known for its ability to infect humans and causing a rapid and mostly fatal primary amoebic meningoencephalitis. When contaminated water enters the nose, the facultative parasite follows the olfactory nerve and enters the brain by crossing the cribriform plate where it causes tissue damage and haemorrhagic necrosis. Although *N. fowleri* has been studied for several years, the mechanisms of pathogenicity are still poorly understood. Furthermore, there is a lack of knowledge on the genomic level and the current reference assembly is limited in contiguity. To improve the draft genome and to investigate pathogenicity factors, we sequenced the genome of *N. fowleri* using Oxford Nanopore Technology (ONT). Assembly and polishing of the long reads resulted in a high-quality draft genome whose N50 is 18 times higher than the previously published genome. The prediction of potentially secreted proteins revealed a large proportion of enzymes with a hydrolysing function, which could play an important role during the pathogenesis and account for the destructive nature of primary amoebic meningoencephalitis. The improved genome provides the basis for further investigation unravelling the biology and the pathogenic potential of *N. fowleri*.

## Introduction

*Naegleria* are free living protists belonging to the family of *Vahlkampfiidae* and occur in fresh warm water sources and soil worldwide. Members of the genus *Naegleria* show a flexible phenotype. Under favourable conditions they exhibit an amoeboid form while formation of fast-moving flagellates or resting cysts is observed in nutrient sparse conditions or during dry periods. Since the first description of *N. gruberi* in 1899, there have more than 40 species been isolated and characterized so far. Despite the huge diversity, only *Naegleria fowleri* is known for its ability to infect humans^1,2^. When contaminated water enters the nose, for example during swimming or ritual nose rinsing, *N. fowleri* overcomes the host’s extracellular matrix (ECM) and follows the olfactory nerves to the brain by crossing the cribriform plate. There the amoeba multiplies, destroys nerve cells and causes primary amoebic meningoencephalitis (PAM) with a mostly fatal outcome^3–5^. Due to the fast progression of the disease, diagnosis is usually made post-mortem by microscopic examination of the cerebral spinal fluid (CSF) or by quantitative PCR^6^. Treatment options are still limited, where the most promising therapy is medication with antibiotics and antifungals such as Amphotericin B, Fluconazole, Rifampicin and Miltefosine^7^. Pathogenicity factors have been investigated for several years but the mechanisms of infection and onset of the disease are still poorly understood. Secretion of proteases and hydrolysing enzymes is known as an important factor during pathogenesis in various eukaryotic parasites. In general, they are involved in the degradation of extracellular matrix proteins, lysis of host cells or invasion of the host cells by intracellular parasites^8–10^. Several studies highlight the importance of proteins with hydrolysing activity in *N. fowleri*. By analysing *N. fowleri* conditioned medium, different cysteine proteases with similarity to cathepsin B have been identified which are most likely involved in the degradation of the ECM and the invasion of the blood-brain barrier^11–15^. In addition, the function of metalloproteinases^16^, phospholipases^17^, elastases^18^ and glucosidases^19^ during invasion of the central nervous system (CNS) have been discussed. Furthermore, secreted proteins are potential drug targets and a recent study examined cysteine protease inhibitors as potential drug against PAM^20^. Although numerous studies describe proteolytic enzymes based on their biochemical properties by using enzyme and proteinase inhibitor assays, the diversity of *N. fowleri* secreted proteins is still sparsely described and the actual genes encoding the factors responsible for the cytotoxic effect remains often unknown. Although the genome of *N. fowleri* (Isolate ATCC 30863) has been published in 2014, the assembly is highly fragmented including over 1,000 contigs and gene annotation on the genomic sequence is missing^21^. Therefore, improving of *N. fowleri* assembly provides the basis for further experiments on a molecular and computational level to unravel the pathogenicity of *N. fowleri*. In the last few years sequencing technologies markedly improved and long-read sequencing using Oxford Nanopore Technology (ONT) offers a new possibility to resolve highly repetitive regions in genome assemblies, leading to highly contiguous reference sequences^22,23^. Nevertheless, one of the major drawbacks of long read sequencing methods is their high error rate. Therefore, hybrid assembly approaches incorporating high quality short-reads are often used to improve the accuracy^23,24^. Recent improvement in sequencing chemistry and base-calling algorithms of ONT lead to an increase in read quality and thus facilitating genome *de novo* assembly and leading to a consensus accuracy of over 99.8%^25^. In this study, we successfully applied ONT sequencing to the genome of the human pathogenic amoeba *N. fowleri ATCC* 30894. The higher contiguity of the *N. fowleri* genome assembly enables the prediction of genes on the nucleotide level using *ab initio* and RNA sequencing (RNAseq) based methods and provides a high-quality reference for further downstream experiments. The secretion of proteins with hydrolysing activity has been discussed as important factor in the pathogenesis of *N. fowleri*. Using deep neural networks implemented in SignalP^26^ and DeepLoc^27^, we identified 208 potentially secreted proteins of which 18% are annotated with a hydrolysing function. In free-living *N. fowleri* they are most likely involved in the lysis of bacterial and eukaryotic microorganism. Given their proteolytic function, these proteins may additionally play a major role in the degradation of ECM proteins and nerve cells and contribute to the pathogenesis of PAM.

## Results

### Genome assembly

To gain a complete overview of the *N. fowleri* genome, total DNA of the isolate ATCC 30894 was extracted and sequenced using ONT. Sequencing of the DNA on the GridION system using one flow cell resulted in a total of 1,352,535 base called reads (9 Gb) with a mean read length of 6,658.6 bp and a N50 of 11,677 bp. The nuclear genome of *N. fowleri* was assembled using the string graph assembler Canu v1.7^28^. To improve consensus accuracy, the initial assembly was polished in two steps using ONT raw signal data by applying Nanopolish v0.11.0^22^ and with high quality Illumina reads by using Pilon v1.22^29^. The final, curated assembly of the nuclear genome consists of 81 contigs with a total length of 29,549,925 bp, the N50 has a size of 717,491 bp while the L50 is 18. Further, the assembly has a GC content of 36.9% which is similar to the previously published isolate *N. fowleri* ATCC 30863 assembly and the *N. lovaniensis* genome, while for *N. gruberi* a slightly lower GC content of 35% is reported. With a size of 717,491 bp, the N50 is 18 times higher than the N50 of the previously published *N. fowleri* ATCC 30863 genome and is comparable to N50 of the *N. lovaniensis* assembly (Table 1).

**Table 1:** Comparison of sequenced Naegleria genomes.

	*N. fowleri ATCC 30894*	*N. fowleri ATCC 30863* ^21^	*N. lovaniensis* ^40^	*N. gruberi* ^39^
**GeneBank accession**	VFQX00000000	AWXF00000000	PYSW00000000	ACER00000000
**Genome Size (Mb)**	29.5	27.7	30.2	40.9
**GC content (%)**	36.9	37	37	35
**Repeat content (%)**	6	2.5	3.5	5.1
**Number of contigs (Scaffolds)**	90	1729 (574)	111	1977 (784)
**N50 (bp)**	717,491	38,128	658,530	159,679
**L50**	18	212	21	68
**Number of predicted genes**	13,925	17,252 (based on RNAseq data)	15,195	16,620

### Assembly quality

**Figure 1:**
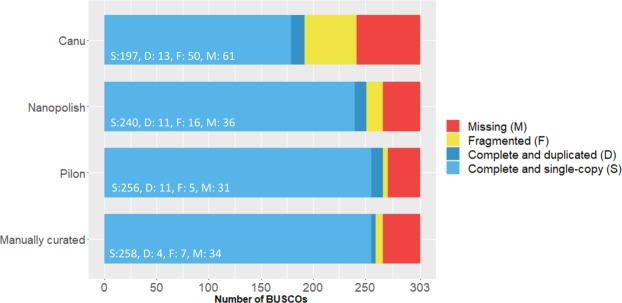
Analysis of Benchmarking Universal Single-copy Orthologs (BUSCOs) at different assembly steps. The genome completeness was evaluated by analysing 303 conserved BUSCOs of the Eukaryota odb9 dataset. The initial Canu assembly contains high numbers of fragmented and missing BUSCOs. Polishing using Nanopolish in combination with ONT raw data and highly accurate Illlumina reads by applying Pilon could reduce the number of fragmented and missing BUSCOs. Further, manual curation using MUMmer alignments and duplicated BUSCOs reduced the occurrence of duplicated BUSCOs.

As measurement for completeness and quality, the presence of 303 Benchmarking Universal Single-Copy Orthologs (BUSCOs)^30^ was analysed at the individual assembly steps. BUSCOs only include genes that have evolved as single copy orthologs over a long time period. Duplication or loss of such genes are considered as rare events, therefore presence or absence of BUSCOs provides an overview of assembly accuracy and completeness^30^. The initial Canu assembly contains 192 complete, 50 fragmented and 61 missing BUSCOs. Polishing using ONT signal level data (Nanopolish) increased the number of complete BUSCOs to 251 while less fragmented (16) and missing (39) BUSCOs are observed. Polishing using high quality Illumina data (Pilon) and manual curation of the assembly further increased the accuracy resulting in 262 complete, 7 fragmented and 34 missing BUSCOs (Fig. 1). Additional rounds of Pilon polishing did not improve the BUSCOs statistics. To benchmark the quality of the ONT assembly, the number of BUSCOs was compared to other previously sequenced *Naegleria* species. Similar numbers of complete BUSCOs are found for the *N. fowleri* ATCC 30863 assembly (264), the *N. lovaniensis* assembly (259) and the *N. gruberi* genome (257). The number of fragmented BUSCOs of the *N. fowleri* ATCC 30894 ONT is slightly higher (7) than observed in the previous Illumina assembly of *N. fowleri* ATCC 30863 (Table 2).

**Table 2:** Analysis of BUSCOs across different sequenced Naegleria species.

	*N. fowleri ATCC 30894*	*N. fowleri ATCC 30863* ^21^	*N. lovaniensis* ^40^	*N. gruber i* ^39^
Complete BUSCOs	262	264	259	257
Duplicated BUSCOs	4	3	16	4
Fragmented BUSCOs	7	3	8	4
Missing BUSCOs	34	36	36	39

### Mitochondrial genome and extrachromosomal sequences

Beside its nuclear genome, *N. fowleri* possesses a mitochondrial genome and encodes its ribosomal subunits on an extrachromosomal plasmid (rDNA plasmid). Due to the high coverage of those elements relative to the chromosomal sequences, when combined, overall assembly performance is poor. Therefore, mitochondrial reads were quality filtered and downsampled using filtlong v0.2.0^31^ and assembled separately using Canu v1.7 followed by polishing using ONT and Illumina reads. The manually curated and circularized mitochondrial assembly has a size of 49,483 bp and sequence comparison to public available reference sequences of strain V419 (Genbank accession KX580903.1) and V212 (Genbank accession NC_021104.1) revealed a similarity of 98.6%. Additionally, reads of the rDNA plasmid were filtered and assembled in the same manner, resulting in a circular contig with the size of 15,936 bp encoding for the small and large ribosomal subunit.

### Genome annotation

First, we annotated the repeats of *N. fowleri. De novo* repeat annotation and masking using RepeatModeler v1.0.11^32^ and RepeatMasker v4.0.8^33^ revealed a repeat content of 6%. Simple repeats represent 1.98% of the repetitive sequences, while 1.85% are LINES and 1.12% are classified as DNA elements. A complete list of repetitive elements is shown in Table 3. Second, genes on the nuclear genome of *N. fowleri* were predicted by an *ab initio* and RNAseq based approach using BRAKER1^34^ while non-coding and ribosomal RNA sequences were annotated by a similarity approach using Infernal 1.1.2^35^ in combination with the Rfam 12.1 database^36^. In total 13,925 genes were predicted and 12,009 (86%) encode at least one PFAM protein domain while to 8,434 (61%) could be annotated with a GO term using BLAST2GO^37^ and to 5,604 (40%) KEGG number could be assigned using eggNOG^38^. Compared to other *Naegleria* species, slightly less proteins were identified: for *N. gruberi* 16,620^39^ genes are reported and in the N., *lovaniensis* assembly 15,195^40^ genes were predicted. Nevertheless, the set of predicted proteins did not show a reduced number complete BUSCOs compared to other *Naegleria* species (Table 4). In addition to the gene coding regions, Infernal 1.1.2 identified tRNAs, and 5 S rRNAs as well as the spliceosomal RNAs U1-U6 on the nuclear genome.

**Tablee 3:** Repetitive elements of the N. fowleri genome.

Repeat Class	Masked length in bp	Percentage of the nuclear genome
SINES	10481	0.04
LINES	544789	1.85
LTR elements	62543	0.21
DNA elements	330505	1.12
Satellites	14240	0.05
Simple repeats	585439	1.98
Low complexity	43175	0.15
Unclassified	205025	0.69

**Table 4:** BUSCO Analysis of predicted proteins across different Naegleria species.

	*N. fowleri ATCC 30894*	*N. fowleri ATCC 30863 (RNAseq based* ^21^ *)*	*N. lovaniensis* ^40^	*N. gruber i* ^39^
Number of Predicted Proteins	13,925	17,252	15,195	16,620
Complete BUSCOs	275	271	270	254
Duplicated BUSCOs	9	11	25	7
Fragmented BUSCOs	4	5	12	14
Missing BUSCOs	24	27	21	35

### Genome similarity

**Figure 2:**
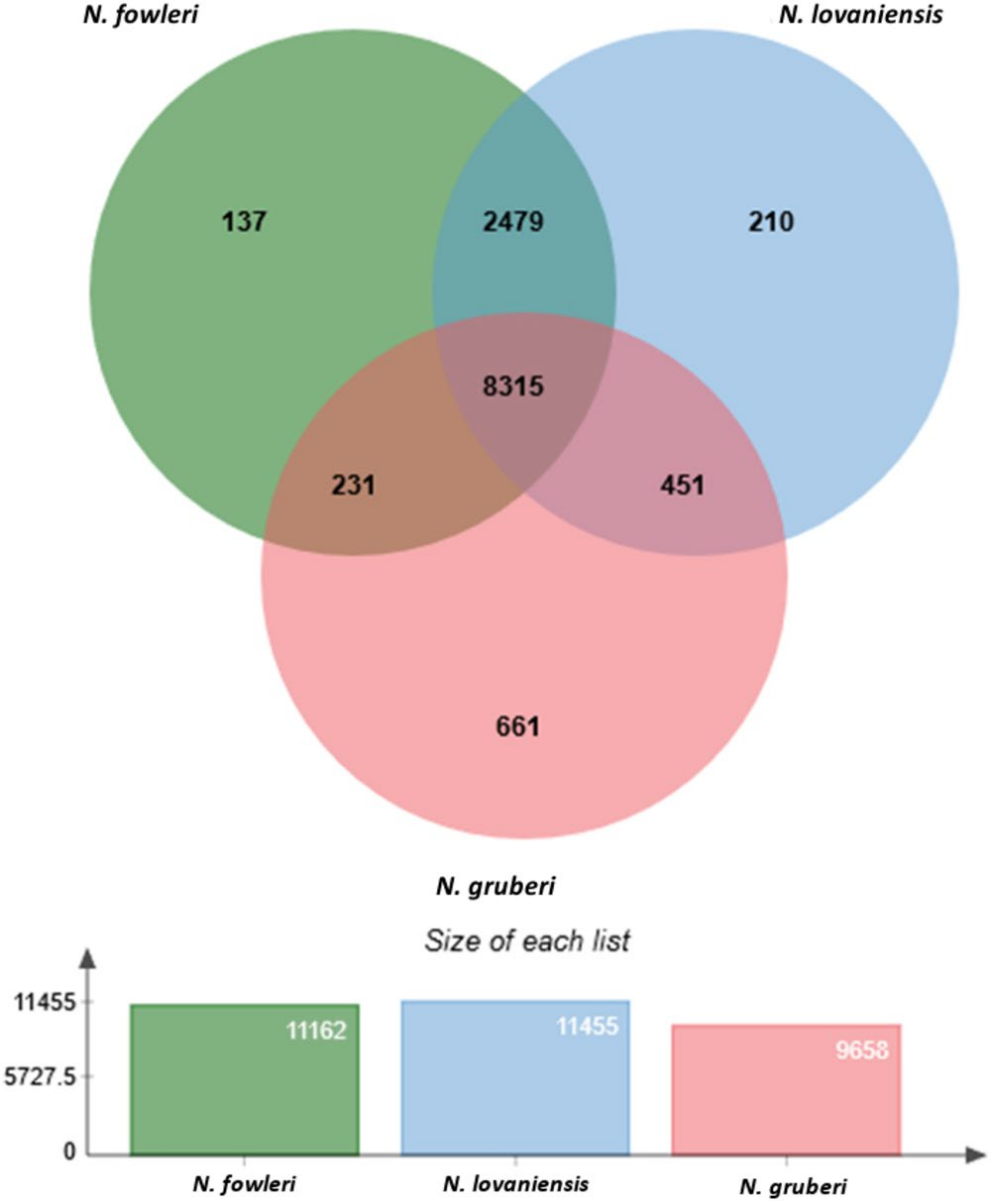
Clustering of predicted *Naegleria* proteins. To gain an overview of the diversity of *Naegleria* species, predicted proteins of *N. fowleri* ATCC 30894, *N. lovaniensis* and *N. gruberi* were clustered using OrthoVenn.

**Figure 3:**
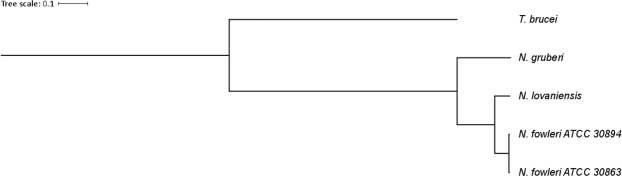
Maximum likelihood tree of sequenced *Naegleria* species. To achieve a comprehensive overview of all *Naegleria* species with available reference genome, a phylogenetic tree was constructed based on maximum likelihood and bootstrapping using RAxML. Evolutionary distances were estimated based on 978 single copy orthologs of all *Naegleria* species and *T. brucei* as taxonomic outgroup.

To gain an overview of the gene repertoire of the genus *Naegleria*, proteins of *N. fowleri*, *N. lovaniensis*, and *N. gruberi* were clustered using orthoVenn^41^. The clustering revealed 8,315 protein clusters shared by all *Naegleria* species. In total, 2,479 clusters are shared between *N. fowleri* and *N. lovaniensis*, while *N. fowleri* shares only 231 protein clusters with *N. gruberi* (Fig. 2). To assess the phylogenetic position of the newly sequenced *N. fowleri* isolate, a phylogenetic tree based on bootstrapping and maximum likelihood was estimated using RAxML v8.2.11^42^ by considering 978 single copy orthologs of the *Naegleria* species with public available genomes. As outgroup we choose the more distantly related *Trypanosoma brucei*. The *N. fowleri* isolate ATCC 30894 is closely related to the isolate ATCC 30863 and *N. lovaniensis*, while *N. gruberi* is more distantly related (Fig. 3).

### Secreted proteins

**Figure 4:**
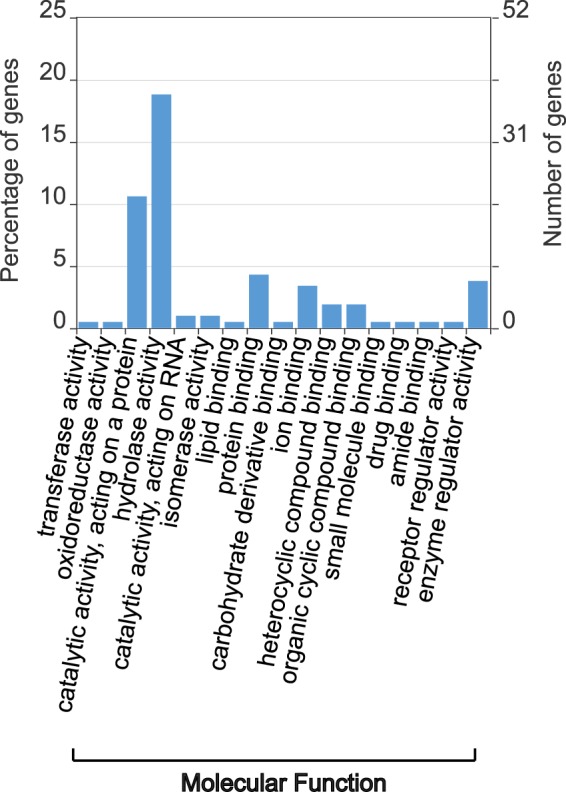
GO annotation of secreted proteins. The abundance of the GO terms within the category Molecular Function was visualized using WEGO. Hydrolase activity is the most abundant category followed by catalytic activity, protein binding and enzyme regulator activity.

**Figure 5:**
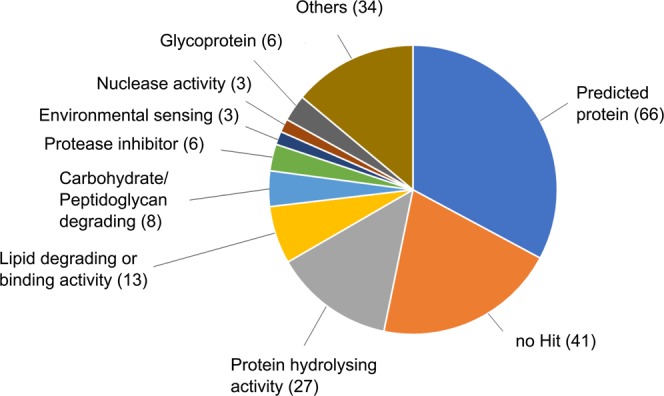
Overview of the function of the secreted proteins. Proteins were classified manually based on BLAST similarity and GO annotation to gain an overview of their biological function. Proteins with a hydrolysing activity are the largest group; in total 27 proteases, 8 carbohydrate/peptidoglycan degrading as well as 13 lipid degrading/binding proteins were identified. 66 proteins have similarities to predicted or uncharacterized proteins, while 41 proteins do not have any similarities to known proteins.

The secretion of proteases and other degradative enzymes are discussed as an important factor during pathogenesis of PAM in order to disrupt the extracellular matrix and nerve cells. To identify proteins secreted by the classical secretory pathway we combined SignalP v5.0^26^ to identify signal peptides and Deeploc v1.0^27^ to verify the cellular localization. In total, we identified  208 potentially secreted proteins of which 163 have BLAST similarities to a protein in the Uniref90. Using the BLAST2GO pipeline v5.2^37^ 75 proteins could be annotated with a GO term. To gain an overview of the molecular function of the secreted proteins, GO annotation were visualized using WEGO v2.0^43^. Almost 20% of the secreted proteins are annotated with the term hydrolase activity (GO:0016787) while 10% are associated with an ion-, protein-, or lipid binding function (GO:0005488). Other terms are catalytic activity (GO:0140096, 10%), enzyme regulator activity (GO:0030234, 3.8%) or isomerase activity (GO:0016853, 1%) (Fig. 4). BLAST similarities and GO annotations were used to further classify the secreted proteins. Regarding proteins with hydrolysing function, 27 proteases including cysteine and serine protases as well as 21 proteins involved in the degradation of lipids and peptidoglycan such as lipases, phospholipases, endolysin like proteins and beta-xylosidases. Additionally, three proteins with similarities to the autocrine proliferation repressor (aprA, Uniprot accession number Q5XM24) and counting complex (countin-1, Uniprot accession number Q86IV5) of *Dictyostelium discoideum* were identified, which suggests the ability of sensing and regulating the population density in *Naegleria*. Additionally, the secretome contains proteins belonging to the Ependymin and Tenascin family as well as different proteinase inhibitors, DnaJ homolog superfamily proteins or ribonucleases. The function of 107 proteins is still unknown. For 41 proteins no BLAST similarity was found and 66 have similarities to predicted or uncharacterized proteins (Fig. 5). A full list of the predicted secreted proteins including their annotation is shown in Supplementary Table S1.

## Discussion

The flexible life stages of *N. fowleri*, including resting cysts, fast moving flagellates and a crawling amoeboid form and its ability to proliferate in fresh water sources as well as a facultative parasite within the host’s CNS makes *N. fowleri* an ideal organism to study fundamental eukaryotic processes and pathogenesis. Although pathogenesis of *N. fowleri* has been studied extensively for more than 50 years, the mechanisms of invasion of the CNS and the ability to survive within the human brain are still poorly understood. Furthermore, the diversity of *N. fowleri* isolates on the genomic level is largely unknown. In this study, we successfully applied ONT sequencing to the genome of the human pathogenic amoeba *N. fowleri* resulting in a highly contiguous reference of the genome comprising of 83 contigs. In comparison to the ONT assembly, the previously sequenced *N. fowleri* ATCC 30863 based on Illumina and 454 sequencing technology is assembled in 1,729 contigs with a N50 of 38,128 bp and L50 of 212 while the total assembly length is 27,791,290 bp. *De novo* assembly using long reads decreased the total number of contigs drastically (90 vs 1729) and lead to an 18 fold increased N50 (717,491 bp vs 38,128 bp). Furthermore, polishing using signal level raw data and high-quality Illumina reads improved the quality of the initial assembly. The final assembly shows similar numbers of complete BUSCOs as other sequenced *Naegleria* species, suggesting that the sequencing and assembly of ONT data resulted in a similar quality and completeness. In addition, the circular sequences of the mitochondrion and the extrachromosomal rDNA plasmid, for which currently no reference is available in public databases, were successfully assembled into single circular contigs. Although less proteins were identified using an *ab initio* approach in the current assembly when compared to other published *Naegleria* genomes, analysis of conserved eukaryotic proteins (BUSCOs) across species did not show a reduced completeness of the protein set. In summary, sequencing and *de novo* assembly of the *N. fowleri* genome resulted in a high-quality draft genome providing the basis for further studies including transcriptomics or proteomics. To gain insight in the biology of the human pathogenic amoeba and to identify factors involved in the pathogenesis of PAM, we predicted 208 potentially secreted proteins and analysed their function based on BLAST similarities and GO annotations. A large proportion of those proteins is annotated with a hydrolysing function but also proteins such as proteinase inhibitors, ribonucleases or enzymes playing a role in sensing of the environment are identified. Hydrolysing enzymes are important factors for accessing nutrients including prokaryotic and eukaryotic microorganism found in soil and fresh water sources. The ability of *Naegleria* species to feed on bacteria as well as on mammalian cells has been described in different studies examining growth conditions in laboratory environments. The addition of heat inactivated bacteria, for example, increases the proliferation rate of *Naegleria* and cultivation on a mammalian cell monolayer has been linked to an increased pathogenicity of *N. fowleri*^44,45^. So far, the genes involved in the assimilation of nutrients are not well studied. By analysing secreted proteins, we identified proteins involved in the degradation of bacterial and eukaryotic cell components. The *N. fowleri* genome encodes for proteins such as endolysin, xylosidases and lipopolysaccharide-binding protein which play a major role in the degradation of bacterial cell membranes that consists of proteoglycan and outer membrane lipopolysaccharides. Further, the protist also secrets phospholipases and ceramidases which enable the hydrolysis of eukaryotic cell membranes. Additionally, 27 secreted proteases were identified which are most likely involved in the degradation of extracellular material. Proteins with a hydrolysing function not only play a role in the nutrition of *Naegleria* but could also serve as potential pathogenicity factors. Among others, the predicted secretome contains proteins which have been previously linked to pathogenicity such as Naegleriapore A (Uniprot accession number Q9BKM2) and virulence-related protein Nf314 (Uniprot accession number P42661). Naegleriapore A was characterized by Herbst *et al*. (2002)^46^ and shows a cytotoxic activity against mammalian and bacterial cells. The virulence-related protein Nf314 is a serine carboxypeptidase and was discovered in 1992 by analysing gene expression patterns in highly and weakly pathogenic *N. fowleri* trophozoites^47^. Other studies highlight the importance of cysteine, serine, and metalloproteases^13,16,48^ as well as of phospholipases^17,49^ during the pathogenesis of *N. fowleri*. However, the actual protein sequences often remain unknown. The here identified proteases and phospholipases are potentially involved in the pathogenesis of PAM and could serve as the missing link between the described proteolytic and lipolytic function and the actual gene sequence. Additionally, proteins belonging to the Ependymin and Tenascin family were identified. Their GO annotations indicate a function in cell adhesion and in binding proteins of the extracellular matrix such as fibronectin or collagen. However, their actual role and how their binding function can be linked to the pathogenicity still has to be examined. Analysis of secreted proteins further shows, that *N. fowleri* secrets DNAJ (HSP40) domain containing proteins which are linked to increased virulence in bacteria and the parasite *Plasmodium falciparum*^50–52^. HSP40 is known as regulator of HSP70^53^, a heat-shock protein which has been linked to pathogenicity of *N. fowleri* previously^54^. Given the regulatory function, it is possible, that HSP40 plays an important role in the pathogenicity of *N. fowleri*. Other proteins of the secretome are protease inhibitors or act as ribonucleases but still little is known about their biological function. The secretion of proteases inhibitors has been described in various parasites including *Apicomplexa*, oomycetes or helminths and a protective function from host proteases is reported for protease inhibitors^55–57^. Further investigations are needed to clarify the role of secreted protease inhibitors in *Naegleria*. Beside proteins with potential involvement in pathogenicity, we found evidence for cell-cell communication and autoregulation of the population density similar as observed in *D. discoideum* by the identification of proteins similar to the autocrine proliferation repressor (aprA) and the counting complex (countin-1).

To conclude, sequencing of the *N. fowleri* genome using long reads resulted in a high-quality draft genome. Furthermore, we identified 208 potentially secreted proteins of which 20% have a hydrolase activity. Given their proteolytic function, they are involved in the lysis of microorganism and eukaryotic tissue cells as nutrients and are therefore considered as potential pathogenicity factors.

## Methods

### Cultivation of *Naegleria and DNA* isolation

To extract high molecular DNA, N. fowleri (ATCC 30894) was cultivated in Nelson’s Medium (pH 6.5)^58^ using Nunclon^TM^ Δ Surface cell culture flasks (Thermo Fisher Scientific, Allschwil, Switzerland). 1 × 10^7^ trophozoites were used for DNA extraction using the DNeasy Blood and Tissue Kit (Qiagen, Basel, Switzerland) according to the manufacturer’s protocol including an RNA digestion step. DNA was finally eluted in 100 μl low TE buffer (10 mM Tris-HCl, 0.1 mM EDTA, pH 8, Thermo Fisher Scientific) and quantified using the Qubit 3.0 Fluorometer (Thermo Fisher Scientific).

### Library preparation and sequencing

1 μg DNA was used for library preparation using the ONT 1D ligation sequencing kit (SQK-LSK109) according to the manufacturer’s protocol without fragmentation step. The resulting library was spotted on a SpotON Flow Cell (FLO-MIN106, R9.4) and sequenced on the GridION X5 sequencer for 48 h. Live base-calling was carried out on the GridION X5 (software release 18.12.1) using ONT MinKNOW 3.1.8 and Guppy v2.0.5 with default options. In addition to the long-read sequencing, 1 μg of the isolated DNA was sent to the Functional Genomics Center Zurich (FGCZ) for sequencing on Illumina NovaSeq. 6000 resulting in 55 Million paired-end reads. For further analysis, Illumina reads were quality trimmed using Trimmomatic 0.36^59^ (options: LEADING:3, TRAILING:3, SLIDINGWINDOW:4:8, MINLEN:36).

### Genome *de novo* assembly and polishing

**Figure 6:**
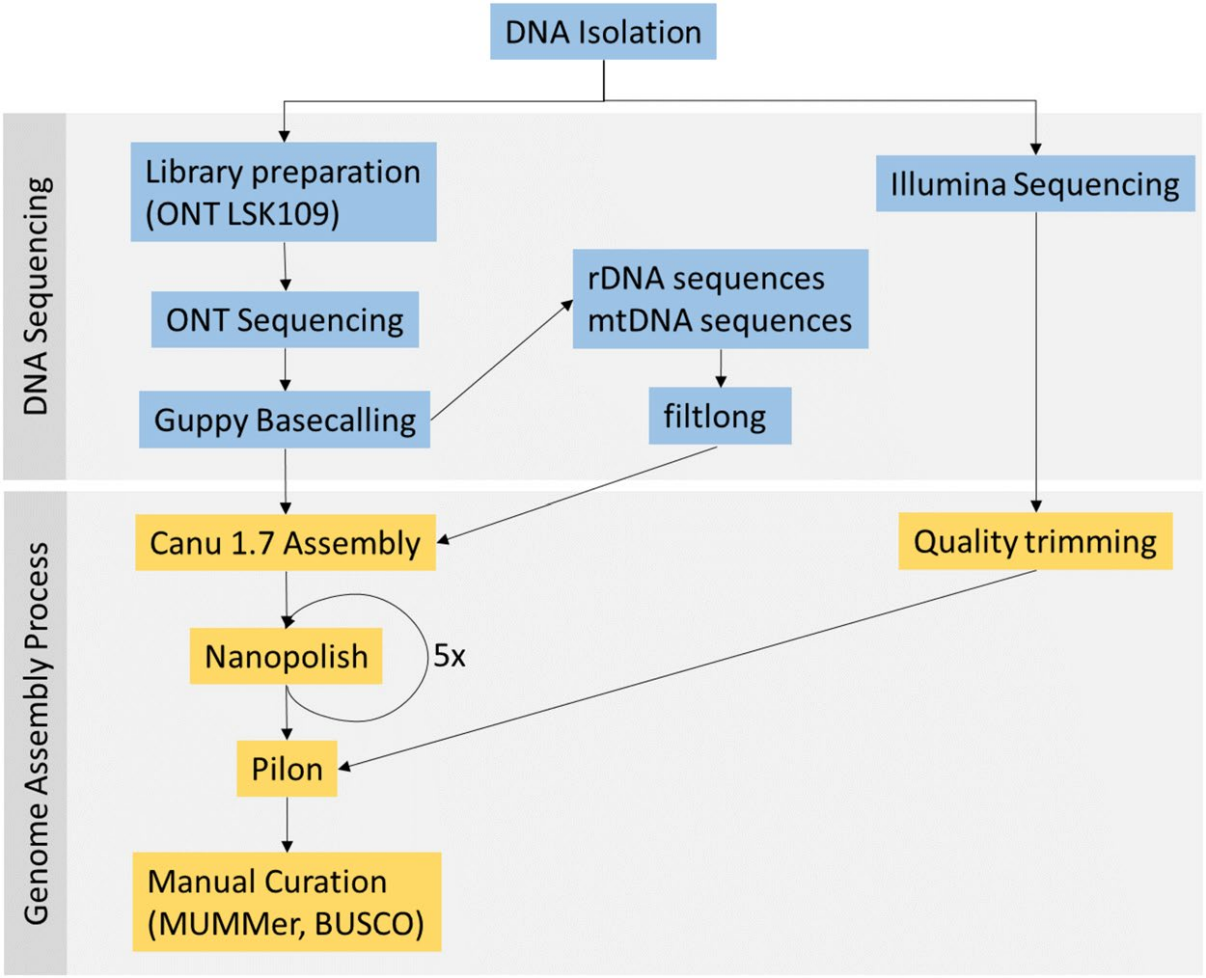
Genome Assembly Workflow. Total DNA was isolated followed by library preparation using the ONT LSK109 kit and sequencing. The other part of the DNA sample was used for Illumina sequencing. Reads of the ONT sequencing were base called using Guppy. Before assembly of the nuclear genome, reads of the mitochondrial genome and the extrachromosomal rDNA plasmid were removed by aligning them to *N. gruberi* reference sequence. The nuclear genome was then assembled using Canu 1.7 followed by 5 rounds of Nanopolish v0.11.0 and one round of Pilon v1.22 in combination with trimmed high-quality Illumina data. The polished assembly was then manually curated to remove redundant sequences based on MUMmer v4.0.0 alignments and duplicated BUSCOs. The mitochondrial genome and the rDNA plasmid were assembled separately. Therefore, reads were quality filtered using filtlong followed by assembly using Canu and polishing using Nanopolish and Pilon as described above.

To facilitate assembly of the genomic sequence, rDNA plasmid reads were excluded from the assembly, by mapping raw reads to the *N. gruberi* rDNA plasmid reference sequence (GenBank accession no. AB298288.1). Unmapped sequences were then *de novo* assembled using Canu v1.7 with default settings. To increase consensus accuracy, raw reads were mapped to the draft assembly using minimap2 v2.8^60^ and Nanopolish v0.11.0^22^ was applied as polishing tool. In total, 5 rounds of Nanoplish were performed to minimize the number of changes. To further improve consensus accuracy, quality trimmed Illumina reads were mapped to the assembly using bwa v0.7.16a^61^ and the sequence was polished using Pilon v1.22^29^. After polishing, the assembly was manually curated to reduce the number redundant contigs by considering MUMmer v4.0.0^62^ all-against-all alignments of the contigs and duplicated BUSCOs. An overview of the sequencing and assembly workflow is shown in Fig. 6.

### Assembly of the mitochondrial DNA and the ribosomal DNA plasmid

Beside its genomic DNA, *N. fowl*eri possesses a mitochondrial genome and a circular plasmid containing the ribosomal sequences, both were assembled separately. To retain 100x coverage of the mitochondrial genome, raw reads were filtered using filtlong v0.2.0^31^ with the parameters*–min_length 1000–keep_percent 90–target_bases 5000000–trim–split 500* and the previously published mitochondrial sequence of *N. fowleri* (GenBank accession no: NC_021104.1) as reference (option *-a*). Filtered reads were assembled using Canu 1.7 followed by polishing using Nanopolish v0.11.0 and Pilon v1.22. Finally, the consensus sequence was manually curated and circularized by aligning to the reference (NC_021104.1). The extrachromosomal rDNA plasmid was assembled in the same manner using the *N. gruberi* rDNA plasmid (GenBank accession no: AB298288.1) as reference. During read filtering using filtlong and options were set to*–min_length 1000–keep_percent 90–target_bases 3200000–trim–split 1000–length_weight 10* to maximize the number of long sequences.

### Repeat annotation

Repeats were *de novo* predicted using RepeatModeler v1.0.11^32^, including RECON^33^ v1.05 and RepeatScout v1.0.5^33^. Sequences with known protein domain were identify by Hmmer3.1b^63^ and the PFAM-A 29.0 database^64^ database and excluded from the library. For further classification into main functional repeat categories, remaining sequences were submitted to TEclass^65^. The curated repeat library was finally used for repeat annotation using RepeatMasker v4.0.8^33^.

### Gene annotation

Gene models were predicted using BRAKER1^34^ in combination with unpublished RNA sequencing data which are part of an on-going project (N. Liechti *et al*., unpublished). Infernal 1.1.2^35^ and the Rfam 12.1 database^36^ were used for annotation of non-coding and ribosomal RNA sequences. PFAM protein domains of de gene coding sequences were identified using HMMER v3.1b2^63^ in combination with the PFAM-A 29.0 database^64^. Sequences were annotated with BLAST^66^ similarity search against UniRef90 with an e-value of 1e-05. Predicted proteins were then annotated using the BLAST2GO v5.2 pipeline^37^ with default mapping parameters including BLAST similarity search against Uniref90 and protein domain search using InterproScan^67^. Additionally, EggNOG mapper^68^ in combination with eggNOG 4.5 orthology data^38^ was used to retrieve KEGG categories. Secreted proteins were predicted by SignalP v5.0^26^ and the resulting set of proteins was additionally analysed for their cellular localization using Deeploc v1.0^27^.

### Genome and proteome completeness

Completeness of the assembly at the individual steps and of the predicted gene models was accessed using BUSCO v3.0.1^30^ in combination with the Eukaryote dataset *odb*9^69^ comprising of 303 single-copy orthologs using the default species parameters. In addition, the number of BUSCOs was compared to previously sequenced *Naegleria* genomes (*N. fowleri* ATCC 30863 (AWXF00000000), *N. lovaniensis* PYSW00000000), *N. gruberi (*ACER00000000)). To assess the completeness of the gene prediction, BUSCO analysis was performed on the BRAKER1 predicted proteins and compared across different *Naegleria* species (*N. fowleri* ATCC 30863 (transcriptome *de novo* assembly^21^), *N. lovaniensis* (predicted proteins^40^), *N. gruberi* (Uniprot reference proteome UP000006671^39^).

### Phylogenetic analysis

To gain an overview of shared orthologs between *Naegleria* species, protein sequences of *N. fowleri* ATCC 30894, *N. gruberi* (Uniprot reference proteome UP000006671^39^), and *N. lovaniensis* (predicted proteins^40^) were clustered using orthoVenn v2.0^41^ with the default parameters (*e-value* = *1e-5*, *inflation value* = *1.5*). Further, phylogenetic relationships were inferred by constructing a maximum likelihood tree. Therefore, the core genome of four *Naegleria* species (*N. fowleri* ATCC 30894 (this study), *N. gruberi*, *N. lovaniensis*, and *N. fowleri* ATCC 30863 (transcriptome based ORFs^21^) and the more distantly related *T. brucei brucei* (Uniprot reference proteome UP000008524) as outgroup was computed using orthoVenn v1.0 (default parameters). The sequences of the resulting 978 single-copy orthologs were aligned using MUSCLE v3.8.31^40^ followed by trimming using trimA v1.4l^70^ and concatenating to a supermatrix using FASconCAT v1.04^71^. The best fitting amino acid replacement model was estimated using Prottest v3.4.2^72^ and the phylogenetic tree was estimated based on maximum likelihood and 1,000 bootstrap iteration using RAxML v8.2.11^42^.

## Supplementary information

Supplementary Table 1

## Data Availability

ONT and Illumina raw reads been deposited at the National Center for Biotechnology Information (NCBI) BioProject repository PRJNA541227 with the accession numbers SRR9047098 (ONT) and SRR9047076 (Illumina). The genome assembly is available under the GenBank accession number: VFQX00000000. The sequences of the predicted proteins are available on figshare (10.6084/m9.figshare.8313656).

## References

[1] Schardinger F (1899). Enwicklungskreis einer Amoeba lobosa (Gymnaamoeba): Amoeba Gruberi. Sitzungsberichte d. kais. Akad. d. Wiss., Abth. 1.

[2] De Jonckheere JF (2014). What do we know by now about the genus Naegleria?. Exp. Parasitol..

[3] Visvesvara GS, Moura H, Schuster FL (2007). Pathogenic and opportunistic free-living amoebae: Acanthamoeba spp., Balamuthia mandrillaris, Naegleria fowleri, and Sappinia diploidea. FEMS Immunol. Med. Microbiol..

[4] Martinez, A. J. & Visvesvaraz, G. S. Free-living, Amphizoic and Opportunistic Amebas. **598**, 583–598 (1875). 10.1111/j.1750-3639.1997.tb01076.x PMC8098488 9034567

[5] Marciano-Cabral F, Cabral GA (2007). The immune response to Naegleria fowleri amebae and pathogenesis of infection. FEMS Immunol. Med. Microbiol..

[6] Bellini NK, Santos TM, da Silva MTA, Thiemann OH (2018). The therapeutic strategies against Naegleria fowleri. Exp. Parasitol..

[7] Grace E, Asbill S, Virga K (2015). Naegleria fowleri: Pathogenesis, Diagnosis, and Treatment Options. Antimicrob. Agents Chemother..

[8] Klemba M, Goldberg DE (2002). Biological Roles of Proteases in Parasitic Protozoa. Annu. Rev. Biochem..

[9] Serrano-Luna J, Piña-Vázquez C, Reyes-López M, Ortiz-Estrada G, de la Garza M (2013). Proteases from Entamoeba spp. and Pathogenic Free-Living Amoebae as Virulence Factors. J. Trop. Med..

[10] Piña-Vázquez C, Reyes-López M, Ortíz-Estrada G, de la Garza M, Serrano-Luna J (2012). Host-Parasite Interaction: Parasite-Derived and -Induced Proteases That Degrade Human Extracellular Matrix. J. Parasitol. Res..

[11] Aldape K, Huizinga H, Bouvier J, McKerrow J (1994). Naegleria fowleri: Characterization of a Secreted Histolytic Cysteine Protease. Exp. Parasitol..

[12] Kim JH (2009). Immunodominant antigens in Naegleria fowleri excretory-secretory proteins were potential pathogenic factors. Parasitol. Res..

[13] Lee J (2014). Novel cathepsin B and cathepsin B-like cysteine protease of Naegleria fowleri excretory-secretory proteins and their biochemical properties. Parasitol. Res..

[14] Coronado-Velázquez D, Betanzos A, Serrano-Luna J, Shibayama M (2018). An *In Vitro* Model of the Blood-Brain Barrier: Naegleria fowleri Affects the Tight Junction Proteins and Activates the Microvascular Endothelial Cells. J. Eukaryot. Microbiol..

[15] Vyas IK, Jamerson M, Cabral Ga, Marciano-Cabral F (2015). Identification of Peptidases in Highly Pathogenic vs. Weakly Pathogenic Naegleria fowleri Amebae. J. Eukaryot. Microbiol..

[16] Lam C, Jamerson M, Cabral G, Carlesso AM, Marciano-Cabral F (2017). Expression of matrix metalloproteinases in Naegleria fowleri and their role in invasion of the central nervous system. Microbiology.

[17] Hysmith RM, Franson RC (1982). Elevated levels of cellular and extracellular phospholipases from pathogenic Naegleria fowleri. Biochim. Biophys. Acta (BBA)/Lipids Lipid Metab..

[18] Ferrante A, Bates EJ (1988). Elastase in the pathogenic free-living amoebae Naegleria and Acanthamoeba spp. Infect. Immun..

[19] Martínez-Castillo M (2017). Nf-GH, a glycosidase secreted by Naegleria fowleri, causes mucin degradation: An *in vitro* and *in vivo* study. Future Microbiol..

[20] Zyserman I (2018). Identification of cysteine protease inhibitors as new drug leads against Naegleria fowleri. Exp. Parasitol..

[21] Zysset-Burri DC (2014). Genome-wide identification of pathogenicity factors of the free-living amoeba Naegleria fowleri. BMC Genomics.

[22] Loman NJ, Quick J, Simpson JT (2015). A complete bacterial genome assembled de novo using only nanopore sequencing data. Nat. Methods.

[23] Tyson JR (2018). MinION-based long-read sequencing and assembly extends the Caenorhabditis elegans reference genome. Genome Res..

[24] Wick, R. R., Judd, L. M., Gorrie, C. L. & Holt, K. E. Completing bacterial genome assemblies with multiplex MinION sequencing. *Microb. Genomics* 0–6, 10.1099/mgen.0.000132 (2017). 10.1099/mgen.0.000132 PMC5695209 29177090

[25] Jain M (2018). Nanopore sequencing and assembly of a human genome with ultra-long reads. Nat. Biotechnol..

[26] Almagro Armenteros, J. J. *et al*. SignalP 5.0 improves signal peptide predictions using deep neural networks. *Nat. Biotechnol*. **37**, (2019). 10.1038/s41587-019-0036-z 30778233

[27] Almagro AJJ, Sønderby CK, Sønderby SK, Nielsen H, Winther O (2017). DeepLoc: prediction of protein subcellular localization using deep learning. Bioinformatics.

[28] Koren S (2017). Canu: scalable and accurate long-read assembly via adaptive k-mer weighting and repeat separation. Genome Res..

[29] Walker BJ (2014). Pilon: an integrated tool for comprehensive microbial variant detection and genome assembly improvement. PLoS One.

[30] Simão FA, Waterhouse RM, Ioannidis P, Kriventseva EV, Zdobnov EM (2015). BUSCO: Assessing genome assembly and annotation completeness with single-copy orthologs. Bioinformatics.

[31] Wick, R. R. filtlong. Available at: https://github.com/rrwick/Filtlong.

[32] Smit, A. F. A. & Hubley, R. RepeatModeler Open-1.0. *RepeatModeler Open-1.0.8*, Available at: http://www.repeatmasker.org (2015).

[33] Bao Z, Eddy SR (2002). Automated de novo identification of repeat sequence families in sequenced genomes. Genome Res..

[34] Hoff KJ, Lange S, Lomsadze A, Borodovsky M, Stanke M (2016). BRAKER1: Unsupervised RNA-Seq-Based Genome Annotation with GeneMark-ET and AUGUSTUS. Bioinformatics.

[35] Nawrocki EP, Kolbe DL, Eddy SR (2009). Infernal 1.0: Inference of RNA alignments. Bioinformatics.

[36] Griffiths-Jones S (2003). Rfam: an RNA family database. Nucleic Acids Res..

[37] Conesa A (2005). Blast2GO: A universal tool for annotation, visualization and analysis in functional genomics research. Bioinformatics.

[38] Huerta-Cepas J (2016). eggNOG 4.5: a hierarchical orthology framework with improved functional annotations for eukaryotic, prokaryotic and viral sequences. Nucleic Acids Res..

[39] Fritz-Laylin LK (2010). The Genome of Naegleria gruberi Illuminates Early Eukaryotic Versatility. Cell.

[40] Liechti N, Schürch N, Bruggmann R, Wittwer M (2018). The genome of Naegleria lovaniensis, the basis for a comparative approach to unravel pathogenicity factors of the human pathogenic amoeba N. fowleri. BMC Genomics.

[41] Wang Y, Coleman-Derr D, Chen G, Gu YQ (2015). OrthoVenn: a web server for genome wide comparison and annotation of orthologous clusters across multiple species. Nucleic Acids Res..

[42] Stamatakis A (2014). RAxML version 8: A tool for phylogenetic analysis and post-analysis of large phylogenies. Bioinformatics.

[43] Ye J (2006). WEGO: A web tool for plotting GO annotations. Nucleic Acids Res..

[44] O’Dell WD, Stevens AR (1973). Quantitative growth of Naegleria in axenic culture. Appl. Microbiol..

[45] John DT (1989). & A., J. R. Cytopathogenicity of Naegleria fowleri in mammalian cell cultures. Parasitol. Res..

[46] Herbst R (2002). Pore-forming polypeptides of the pathogenic protozoon Naegleria fowleri. J. Biol. Chem..

[47] Hu WN, Kopachik W, Band RN (1992). Cloning and characterization of transcripts showing virulence-related gene expression in Naegleria fowleri. Infect. Immun..

[48] Serrano-Luna J, Cervantes-Sandoval I, Tsutsumi V, Shibayama M (2007). A biochemical comparison of proteases from pathogenic Naegleria fowleri and non-pathogenic Naegleria gruberi. J. Eukaryot. Microbiol..

[49] Fulford DE, Marciano-Cabral F (1986). Cytolytic Activity of Naegleria fowleri Cell-free Extract. J. Protozool..

[50] Takaya A, Tomoyasu T, Matsui H, Yamamoto T (2004). The DnaK/DnaJ Chaperone Machinery of Salmonella enterica Serovar Typhimurium Is Essential for Invasion of Epithelial Cells and Survival within Macrophages, Leading to Systemic Infection. Infect. Immun..

[51] Neckers L, Tatu U (2008). Molecular Chaperones in Pathogen Virulence: Emerging New Targets for Therapy. Cell Host Microbe.

[52] Hiller NL (2012). A Host-Targeting Signal in Virulence Proteins Reveals a Secretome in Malarial Infection. Science (80-.)..

[53] Fan CY, Lee S, Cyr DM (2003). Mechanisms for regulation of Hsp70 function by Hsp40. Cell Stress Chaperones.

[54] Song KJ (2008). Heat shock protein 70 of Naegleria fowleri is important factor for proliferation and *in vitro* cytotoxicity. Parasitol. Res..

[55] Pszenny V (2000). Molecular cloning, sequencing and expression of a serine proteinase inhibitor gene from Toxoplasma gondii. Mol. Biochem. Parasitol..

[56] Tian M, Huitema E, Da Cunha L, Torto-Alalibo T, Kamoun S (2004). A Kazal-like extracellular serine protease inhibitor from Phytophthora infestans targets the tomato pathogenesis-related protease P69B. J. Biol. Chem..

[57] Ranasinghe SL, McManus DP (2017). Protease Inhibitors of Parasitic Flukes: Emerging Roles in Parasite Survival and Immune Defence. Trends Parasitol..

[58] Weik RR, John DT (1977). Agitated Mass Cultivation of Naegleria fowleri. J. Parasitol..

[59] Bolger AM, Lohse M, Usadel B (2014). Trimmomatic: A flexible trimmer for Illumina sequence data. Bioinformatics.

[60] Li, H. Minimap2: pairwise alignment for nucleotide sequences. *Bioinformatics* 1–7, 10.1093/bioinformatics/bty191 (2018). 10.1093/bioinformatics/bty191 PMC6137996 29750242

[61] Li H, Durbin R (2009). Fast and accurate short read alignment with Burrows-Wheeler transform. Bioinformatics.

[62] Marçais G (2018). MUMmer4: A fast and versatile genome alignment system. PLOS Comput. Biol..

[63] Eddy SR (2011). Accelerated Profile HMM Searches. PLoS Comput. Biol..

[64] Finn RD (2014). Pfam: The protein families database. Nucleic Acids Res..

[65] Abrusán G, Grundmann N, DeMester L, Makalowski W (2009). TEclass - a tool for automated classification of unknown eukaryotic transposable elements. Bioinformatics.

[66] Altschul S, Gish W, Miller W (1990). Basic local alignment search tool. Journal of molecular biology.

[67] Jones P (2014). InterProScan 5: genome-scale protein function classification. Bioinformatics.

[68] Huerta-Cepas J (2017). Fast Genome-Wide Functional Annotation through Orthology Assignment by eggNOG-Mapper. Mol. Biol. Evol..

[69] Simão, F. A., Waterhouse, R. M., Ioannidis, P., Kriventseva, E. V. & Zdobnov, E. M. Eukaryota Dataset Odb9. Available at: Busco.ezlab.org/datasets/eukaryote_odb9.tar.gz. (Accessed: 4th November 2016).

[70] Capella-Gutiérrez S, Silla-Martínez JM, Gabaldón T (2009). trimAl: A tool for automated alignment trimming in large-scale phylogenetic analyses. Bioinformatics.

[71] Kück P, Meusemann K (2010). FASconCAT: Convenient handling of data matrices. Mol. Phylogenet. Evol..

[72] Darriba D, Taboada GL, Doallo R, Posada D (2011). ProtTest-HPC: Fast Selection of Best-Fit Models of Protein Evolution. in Lecture Notes in Computer Science.

